# Traumatic Ulceration of the Vestibular Mucous Membrane After Insertion of Four Mini-Implants in the Atrophied Mandible: A Case Report

**DOI:** 10.1155/2024/4662194

**Published:** 2024-10-24

**Authors:** Domagoj Kilić, Sanja Peršić-Kiršić, Asja Čelebić

**Affiliations:** ^1^School of Dental Medicine, University of Zagreb, Gundulićeva 5 10000, Zagreb, Croatia; ^2^Department of Removable Prosthodontics, School of Dental Medicine, University of Zagreb, Zagreb, Croatia

**Keywords:** atrophied mandible, complication, decubital ulcer, dentistry, increased perioral muscle tonus, mini-implants, oral surgery, silicone splint, vestibular movable mucous membrane

## Abstract

This clinical report describes how a decubital ulcer arose from the direct contact of the vestibular movable mucous membrane against mini-implant balled-type heads after the mini-implant insertion in the edentulous atrophic mandible of a 78-year-old patient who was not wearing a conventional mandibular complete denture for more than 10 years. Due to severe alveolar ridge atrophy, mini-implant insertion (2.0 mm wide) was an option without extensive surgical procedures. The patient signed the informed consent. A few days after the implant insertion, injury, inflammation, and induration of the vestibular movable mucous membrane were observed on the movable vestibular mucosa on the right side, opposing the mini-implants. The cause of inflammation was attributed to increased perioral muscle tonus which pushed the movable mucosa onto the mini-implant heads and caused mechanical trauma. During the period of edentulism, the perioral muscle tonus increased, directing the mucous membrane of the lips and cheeks against residual ridge to enable food comminution. To treat the persistent decubitus, a bulk of dental composite resin was placed around mini-implant heads and light-cured to protect the mucosa from further mechanical trauma, as the patient did not possess an old mandibular denture to cover the mini-implant heads. Vestibuloplastic surgery (disinsertion of movable attachments and deepening of the vestibulum) was also done. After the surgery, a silicone splint, resembling an occlusal rim, was made to protect the mucous membrane, keep medicaments for faster epitalization in place, to decrease perioral muscle tonus before the new dentures' delivery, and to prevent movable tissue relapse. The custom impression, jaw relationship determination, and try-in of the artificial teeth setup were made with sutures still in place. After the denture delivery and implant loading, the patient was instructed to sleep with the dentures to protect the movable mucous membrane. One year later, almost no peri-implant marginal bone loss was observed, attached and peri-implant mucosa were healthy, and the patient was delighted.

## 1. Introduction

Mini dental implants (MDIs) with a surface prepared for osseointegration (SLA) have been used for over 20 years, representing an alternative treatment to bone augmentation and standard-sized implant insertion in patients with narrow alveolar ridges [1–3]. In very old edentulous patients, especially in the long-time complete-denture wearers with ill-fitting dentures, the alveolar ridge can be considerably reduced, mostly due to local factors [4, 5]. Sometimes, the ridge atrophy can be so advanced that only extensive surgical and augmentative procedures can enable the placement of implants of a standard size diameter.

Mini-implant insertion may be an alternative therapy to two standard-size implants for mandibular overdenture retention, especially in geriatric patients. MDIs are listed in Category 1 of narrow implants (diameter ranging from 1.8 to 2.5 mm) [6]. Their insertion can often be obtained without raising a flap, thus decreasing the duration of postsurgical recovery and pain [7]. The insertion of four MDIs for mandibular overdenture retention has been verified as an excellent treatment option due to excellent survival and success rates and increased patient satisfaction [8–12]. Even short MDIs (6.0–8.0 mm long) can be successfully used in extreme mandibular atrophy [13, 14]. Sometimes, even only three MDIs (10–14 mm long) can successfully retain a mandibular overdenture for a long period [15]. Furthermore, it has been reported that MDIs represent even a successful treatment option for the retention of removable partial dentures [16–19], for support of crowns and short bridges in the mandibular incisor region [20], for support of crowns in the second incisor maxillary region [21], for mandibular overdenture retention in children with ectodermal dysplasia [22], and retention of mandibular resection prosthesis [23]. However, MDIs showed less successful outcomes when they were used for retention of maxillary overdentures [24, 25].

Some complications with MDIs have been described in the dental literature, such as implant fractures, early implant loss, insufficient implant primary stability, or peri-implant bone loss over time [2, 3, 10, 12]. Technical complications with overdentures (denture fractures, artificial tooth loss) or with attachments (need for resilient “o” ring or metal housing replacement) have also been reported [13, 15, 16, 26–30]. However, to our best knowledge, no data exists reporting an occurrence of vestibular decubital ulcerations after the insertion of MDIs in the mandible. Only one case of decubital ulceration of peri-implant mucosa in the sublingual fold after the insertion of four MDIs has been reported and assigned to a reduced attached mucosa and distortion of movable mucosa by the tongue muscles against the 2.0-mm-wide mini-implant heads [31].

## 2. Clinical Report

### 2.1. Diagnosis and Etiology

**Figure 1:**
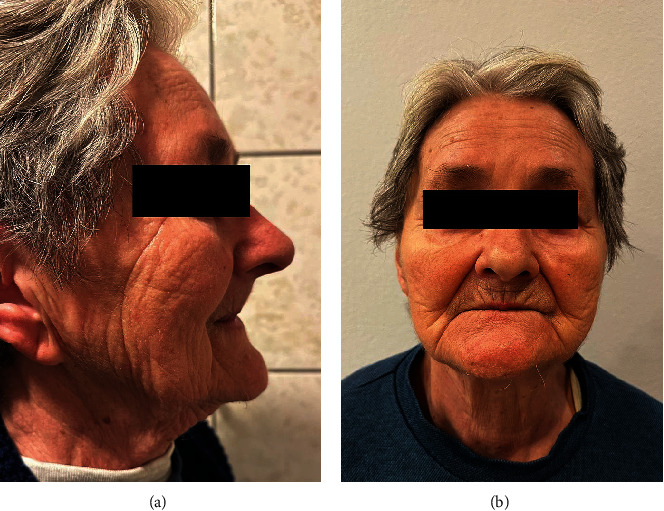
Typical sunken facial appearance of the patient who was not wearing a mandibular complete denture for 10 years: (a) lateral view, (b) frontal view.

**Figure 2:**
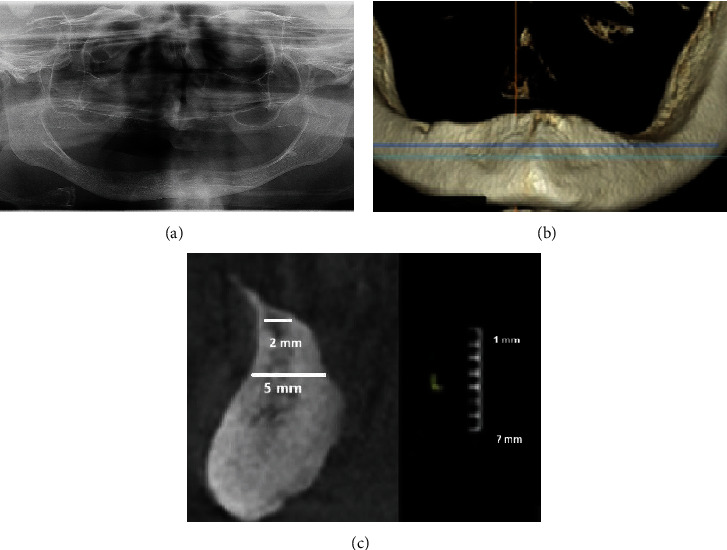
Preoperative radiological images: (a) panoramic radiogram, (b) CBCT shows a pointed top of the alveolar ridge, and (c) a sagittal view of the mandible with a gauge presenting the amount of leveling (osteotomy) that would be necessary to obtain the 5-mm width.

A 78-year-old woman, not wearing a conventional mandibular denture for more than 10 years and having problems with food regurgitation and spasms of the esophagus asked for treatment. The clinical examination revealed that she had a typical collapse of the lower third of the face, the “sunken and aged” facial appearance with the lips sagged and twisted inwards (Figures 1(a) and 1(b)). The digital panoramic radiogram (Soredex Scanora software 2D, Tuusula, Finland) (Figure 2(a)) showed advanced bone atrophy. The clinical examination also revealed a pointed mandibular ridge confirmed by the CBCT scan (Soredex Scanora software 3D, Tuusula, Finland) (Figures 2(b) and 2(c)). Additionally, unfavorable movable tissue attachments very close to the narrow area of the keratinized attached mucosa were also detected.

### 2.2. Treatment Objectives

The treatment objectives were to rehabilitate the patient by restoring her chewing function and orofacial aesthetics and to increase her oral health-related quality of life (OHRQoL). Among different possible therapies, which were manufacture of conventional dentures after the vestibuloplastic surgery; or bone levelling/augmentation (osteotomy with possible interposition of autologous bone or bone substitute) along with disinsertion of soft tissues plus insertion of standard-width implants to support a removable overdenture; or “all-on-4” treatment; or custom-made periosteal implants, or mini-implants), the patient decided for the insertion of four mini-implants in the mandibular interforaminal region to support a new complete overdenture and for manufacturing of a new maxillary conventional complete denture. It was explained to the patient that the vestibuloplasty and movable tissue de-attachment in the vestibulum of the mandible could also be an option along or after the mini-implants' insertion.

### 2.3. Treatment Alternatives

One of the treatment alternatives was the manufacture of a new conventional complete mandibular (after the vestibuloplastic surgery) and maxillary conventional removable complete denture. Other treatment options were the insertion of Category 2 or 3 two-piece narrow implants or even the insertion of two implants of standard-size diameter along with leveling of the residual ridge to the desired width for implant placement. However, leveling (osteotomy) and removal of the crest of the ridge to obtain sufficient width might also include disinsertion of tissues in the sublingual fold, or in case of a less aggressive osteotomy, the interposition of a bone substitute might be needed, and a pause of at least 4–6 months after grafting. Custom-made subperiosteal implants could also be considered as an alternative to regenerative procedures for the rehabilitation of severe bone atrophy, or an “all-on-4” treatment for fixed full-arch rehabilitation of the edentulous mandible, which also might have included severe bone levelling and/or bone augmentation for the two anterior implants.

### 2.4. Treatment Progress

**Figure 3:**
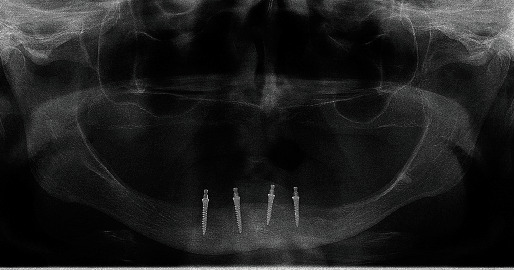
Panoramic radiogram after insertion of four MDIs.

The patient chose the insertion of four 2.0-mm-wide MDIs in the mandible to retain the mandibular overdenture and a conventional maxillary denture. After signing the informed consent and after the Ethical Committee approval (no. 05-PA-26-6/2015), the open-flap surgical procedure was performed for the MDI insertion. The patient took 2 g (prophylactic dose) of antibiotic medication (Amoxicillin Belupo, Belupo lijekovi i kozmetika d.d., Koprivnica, Croatia) 1 h before the surgery. Using a physio-dispenser (W&H Implantmed, GmbH, Austria) with an external drill cooling and under local anesthesia (Ubistesin Forte 4%, 3M, GmbH, Germany), the pointed top of the ridge was removed only to obtain sufficient width for the 2-mm-wide implants. The bone was levelled to a width of at least 3.5 mm. The bed of the entire implant length was prepared in the bone using a smaller diameter drill (1.5 mm) than the implants' width (2.0 mm). Four MDIs (Dentium, Seoul, South Korea) were inserted in the mandibular interforaminal region in positions previously occupied by the left and right premolars and the left and right second incisors. During insertion, the MDIs were rotated clockwise until the whole roughened threaded surface was in the bone (dimensions of the MDIs on the right side were 2.0 mm wide and 10.0 mm long, and on the left side 2.0 mm wide and 8.0 mm long). The final insertion torque values varied between 35 and 50 N/cm, presenting a satisfactory primary stability. After suturing, a new panoramic radiogram was obtained to control the positions of the inserted MDIs (Figure 3). The sutures were removed after 9 days.

### 2.5. Treatment Results

**Figure 4:**
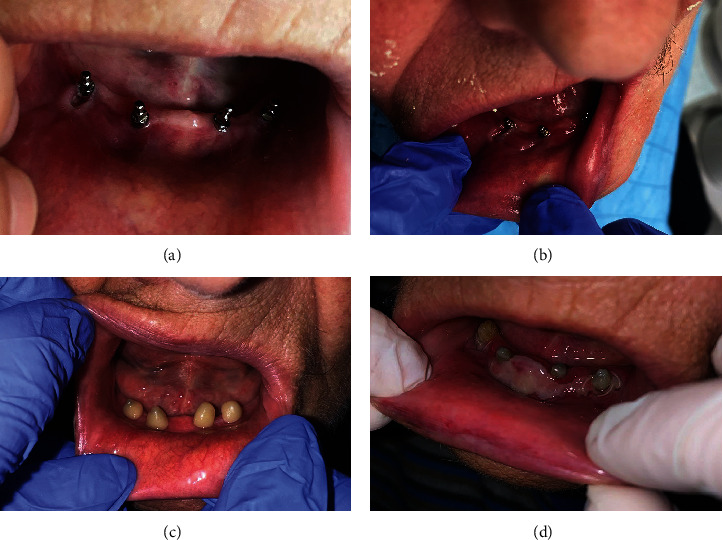
Sequence of inflammation, protection of the movable mucous membrane with a bulk of composite, and the result of the vestibuloplastic surgery. (a) Inflammation of the mechanically irritated movable mucous membrane opposite the mini-implants on the right side of the mandible and insufficient amount of keratinized attached mucosa in the vestibulum. (b) Imprints of the mini-implant balled-type heads in the vestibular mucous membrane. (c) Composite material covering the mini-implants to prevent further injuries of the movable tissues. (d) The second day of healing after Clark's vestibuloplasty. (a–d) Movable tissues of the sublingual fold are attached very close to the crest of the ridge.

**Figure 5:**
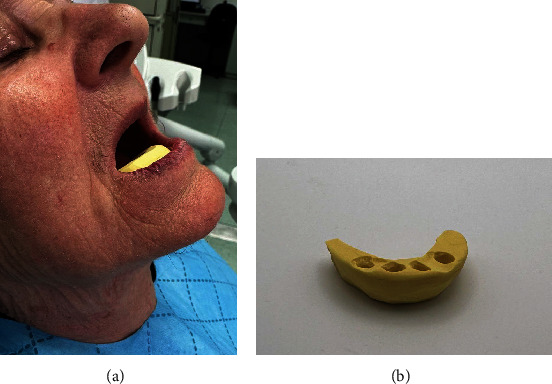
A temporary splint made of a putty C-silicone: (a) splint placed in a mouth and (b) splint outside the mouth.

**Figure 6:**
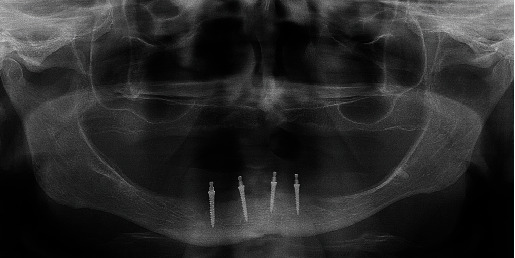
The control panoramic radiogram 1 year after loading of mini-implants.

**Figure 7:**
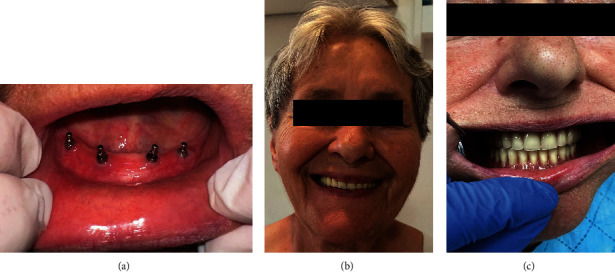
The control clinical examination at the 1-year follow-up. (a) Healthy tissues with a sufficient area of keratinized mucosa around implants and sufficient depth of the vestibulum. (b) Satisfied patient smiling after prosthetic rehabilitation. (c) Frontal view of the occlusion.

A week after suture removal, the patient complained of pain originating from the inner mucous surface of her lip and cheek-facing implants on the right side. The pain was intensifying during tissue movements and chewing. The mucosa showed a deep red inflammatory color (Figure 4(a)). Since a bacterial infection was suspected of the injured mucosa, antibiotic therapy (Klavocin 1 g, Pliva, Zagreb, Croatia, and Medazol 400 mg, Belupo d.d., Koprivnica, Croatia) was prescribed. Injuries were probably elicited by a high muscular tonus on the right side of the mandible pushing the movable tissue onto the mini-implant heads, as well as insufficient area of keratinized peri-implant tissue. It was not possible to cover the MDIs with the old denture as the patient did not possess it. A week later, there was no evidence of healing, and the inflammation got even worse. Decubital injuries opposite the MDI heads in the positions of the previous first right premolar and second right incisor were detected (Figure 4(b)). The mucous membrane was swollen, hard, and indurated. To stop the progression of this mechanical irritation, a thick layer of composite resin (Tetric line, Ivoclar, Lichenstein) was placed around each implant ball-type head, light-cured, and polished (Figure 4(c)). The next day, Clark's vestibuloplasty [32, 33] was also performed to increase the vestibulum depth and to achieve a larger area of the attached keratinized mucosa (Figure 4(d)). A horizontal incision was made over the top of the ridge between implants trying to preserve the periosteal layer. The mucosa was undermined and sutured with resorbable sutures (PGA 4-0, Meiyi, Huaiyan medical instruments, China). Additionally, on the second day after the vestibuloplastic surgery, a horseshoe-shaped splint of high-viscosity silicone material (Optosil putty, Heraeus Kulzer, GmBH, Germany) was made. It was done after obtaining the alginate impression (Chroma Fast, Kulzer GmbH, EU) of the denture-bearing area. The cast was poured in a plaster (Polistone, Polident d.o.o., Volčja Draga, Slovenia), and the vestibular surface of the residual ridge was covered with a 1-mm-thick layer of pink wax (modeling wax sheets, Carmel industries, Guajart, India) to leave space between the vestibular surface and the splint's flange for application of medicaments for faster epithelization after the vestibuloplasty. The putty (Optosil putty, Heraeus Kulzer, GmBH, Germany) was mixed, placed over the cast, and shaped to resemble a wax rim for bite registration in edentulous patients. Gengigel (Ricerfarma, Milan, Italy) was applied at the inner surface of the splint ensuring faster epithelization and wound protection, covering the surgically treated area (Figures 5(a) and 5(b)). The custom impressions for final dentures were obtained 11 days after the vestibuloplastic surgery using a custom light-curing acrylic tray (Huge Dental Material Corporation, China), thermoplastic material for border moulding (Iso Functional, GC, Tokyo, Japan), and a polyether impression material (Impregum penta super quick, 3M ESPE). At that time, the sutures were still in place, but the epitelization was sufficient for obtaining the impression. The master casts were poured into the hard stone (Zhermack Elite, Type 4, Zhermack GmbH, Germany), occlusal rims were made, the jaw relationships were determined in the centric position, and the face-bow transfer was done. Artificial teeth (Ref-Line, Polident, Volčja Draga, Slovenia) were set in the articulator (SAM 2 PX, GmbH, Germany) and checked in the mouth for aesthetical approval. Three weeks after the vestibuloplastic surgery and 2.5 months after the MDIs' insertion, new dentures were delivered to the patient and implants were loaded by the “o”-ring attachments in metal housings (Dentium, Seoul, South Korea). The ulceration and induration of the mucous membrane completely disappeared at the time of the overdenture loading. The patient was ordered for the control examination and denture adjustments 14 days after the surgery. After 1 year with the dentures in the patient's mouth, the control panoramic radiograph revealed almost completely stable peri-implant marginal bone (Figure 6). Healthy peri-implant tissues with an absence of inflammation of the mucous membrane on the inner surface of the lips and cheeks and sufficient vestibular depth were observed (Figure 7(a), 7(b), 7(c)). The patient was very satisfied and reported that even food regurgitation and esophagus spasms were significantly reduced after successful prosthetic rehabilitation. An overview of all procedures, treatment steps, and results of the therapy are summarized in Table 1.

## 3. Discussion

Rehabilitation with complete mandibular dentures or overdentures is always challenging. The two standard-sized implants, which are recommended as the minimum therapy for the edentulous mandible [34], sometimes cannot be inserted in patients with reduced alveolar ridge width without demanding procedures. Four implants of narrower diameter represent an alternative treatment, but sometimes, in the narrowest ridges, only Category 1 narrow implants, that is, MDIs, can be inserted without augmentation procedures. However, there are various possibilities for treating severely atrophied mandibles with or without bone augmentation [35–38]. The patient in this study refused bone augmentation or any other extensive surgical treatment options, as she was 78 years old, and she did not want to wait long for the treatment results but preferred the fastest options. The cost of the treatment was also a problem for her, as she was not able to afford more expensive treatment modalities. Therefore, she decided for the insertion of four mini-implants to retain her new mandibular overdenture, as it was the fastest and the least expensive treatment. She refused the treatment with the new conventional mandibular denture because she was convinced that she would not be able to wear it due to her 10-year previous experience.

She needed an open-flap surgery even for the 2.0 -mm-wide MDI insertion, as the pointed top of the residual ridge was too thin and had to levelled to the 3.5 mm. However, it could have been possible for further levelling to at least 5 mm in diameter, thus allowing standard therapy (two standard-width implants), but the patient preferred four MDIs, mostly due to the lower cost. Also, the Specialist in Prosthodontics thought that less bone levelling would leave some space for a denture flange in the sublingual space before reaching movable tissues, as the sublingual fold was already shallow (Figures 2(c) and 4(a), 4(b), 4(c), 4(d)).

The whole mini-implant length had to be drilled in the bone instead of the recommended two-thirds, but with a narrower drill to achieve sufficient primary stability [13, 39–41]. The MDIs ended in a very dense bone and could fracture under too high insertion torque; therefore, the drilling encompassed the whole implant's length [13, 14]. Although good primary stability was achieved, immediate loading of the implants was not done because the patient did not possess an old conventional complete mandibular denture, which was lost during the 10 years of not wearing it.

After the MDIs were inserted, decubital injuries, inflammation, and induration of the inner surface of the lip and cheek mucous membrane appeared, probably due to the increased tonus of perioral muscles and mechanical trauma of the movable mucous membrane. During the 10 years of not wearing a mandibular denture, the patient's perioral muscle tonus increased due to assisting in a masticatory process by pressing the lip's and cheek's mucous membrane inwards, against the residual ridge, to enable food diminution. After implant insertion, although placed in the center of the levelled residual ridge (refers to buccolingual width), the movable mucous membrane was pressed against the MDI heads by a strong force of increased muscular tonus. Although rounded, heads are also thin, thus making it possible to injure the membrane under strong pressure. The MDIs were inserted in the center of the thin residual ridge in the zone which should be “neutral.” However, it was not neutral due to the increased muscle tonus; therefore, the MDI heads were “an obstacle” to acquired tissue movements pushing the mucous membrane towards the ridge. The “imprints” of the MDI heads in the mucous membrane even existed (Figure 4(b)). Since the injury did not heal under antibiotic therapy (at first glance, bacterial superinfection was suspected), we were aware that we are not dealing with a secondary bacterial infection, but rather with the mechanical trauma.

The mucosal tissues of the cheeks and lips are normally supported by teeth. In edentulous patients, such support is lost, leading to decreased vertical dimension of the lower third of the face. In such circumstances, perioral and masticatory muscles change the pattern of activity by decreasing or increasing their tonus to enable chewing [42, 43]. One study reported that the increased EMG activity decreased 3 months after the insertion of a new prosthesis [44]. The new dentures enabled the adaptation of muscle tonus throughout 1 year of denture utilization [43, 45]. The deteriorated appearance of the patient was also substantially improved after treatment [46].

The vestibuloplasty was necessary to deepen the vestibulum for the denture flange and to ensure sufficient width of the attached peri-implant keratinized mucosa. It was done in the second surgical session due to the complexity of both procedures. Except for pain, a disadvantage of the applied technique is the unpredictable relapse of movable tissue [32]; therefore, the sutures were removed only at the denture delivery and implant loading, 3 weeks after the procedure. A temporary silicone splint resembling an occlusal rim served to keep medicaments in place for faster epithelization, to adjust the perioral muscle tonus, to protect the lip and vestibular mucosa from further injury, and to prevent relapse of movable tissue. The Gengigel (which contains a hyaluronic acid) was used for faster wound healing. However, some other methods and products could also be used to improve and speed up wound healing, such as ozone [47], photobiomodulation [48], probiotics [49], platelet-rich fibrin [50], and oral disinfecting solutions, such as chlorhexidine [51].

The therapy with four MDI-retained mandibular overdenture was chosen because the patient refused extensive treatment procedures and because in vitro studies [52] revealed good MDI behavior considering stress distribution during denture loading. Stress distribution and extent are very important in narrow implants. Maximum von Mises stress both in standard and mini-implants occurs at the neck of the implants [52]. In narrow ridges, MDIs are usually surrounded by a cortical bone, which is of D1 density, thus offering good mechanical support. The stress and strain values in the mandible are 68.15% higher with two mini-implants than with two standard implants [53]. Peri-implant microstrains appear to be lower when four or three MDIs are used than with only two MDIs [54–56], which is one of the reasons for the insertion of four MDIs. Warin et al. [57] concluded that the use of a low number of MDIs tends to produce low strain values in the retromolar denture-bearing area and around the terminal MDIs during posterior loadings, but when using a high number of MDIs, the overdenture tends to have more stability during function.

The ball attachments, chosen for the mandibular overdenture retention in this study, demonstrate lower stress within the implants compared to those with magnet attachments under vertical and oblique loadings [52, 53]. It has also been proved that both standard-sized or mini-implants increase chewing forces when used to retain and support a mandibular overdenture [58]. Clinical studies, which now cover periods from 1 to 10 years of clinical utilization also revealed good survival rates of mini-implants and increased patient satisfaction [1–3, 7–19, 23–31]. Increased patient satisfaction and OHRQoL reported in this study is in line with other publications showing that implant or mini-implant-supported overdentures significantly increase OHRQoL, chewing function, and orofacial aesthetics [19, 59–65].

Although sometimes a patient's and a therapist's opinion may be different concerning the outcome of therapy and the patient may remain unsatisfied [66–71], when we have a cooperative patient, even in the event of complications that need additional interventions, the most complex and demanding situations can be resolved by mutual satisfaction, as it was in the case of the presented patient.

The study's limitation is that we did not obtain mucosal tissue samples for histologic analysis. Only one clinical report showing similar mechanical complications located in the sublingual fold [31] described the lesion as having whitish areas focally, surrounded by erythema with central focal ulceration, topographically associated with the mini-implant heads. Their histological specimens showed a reactive hyperplastic epithelium adjacent to the ulceration covered by a fibrinopurulent membrane composed of granulation tissue and numerous small vessels. Within the inflammatory component, a dominant population of eosinophils was found, dispersed throughout the lesion, while lymphocytes, scarce histiocytes, and rare atypical cells were also present. The lesion was listed as a traumatic ulcerative granuloma with stromal eosinophilia (TUGSE), which is a benign, rapidly growing ulcerative lesion. Trauma was an important contributing factor. The lesion was surgically removed in that case [31]. However, for the patient described in this study, sample probing for histological analysis or excision was not done because when the mechanical trauma was eliminated, the wound healed completely. In the future, if a similar problem ever occurs, it would be interesting to obtain a sample for histologic analysis and to test other preventive therapies in wound healing.

## Figures and Tables

**Table 1:** A brief overview of procedures, treatment steps, and results of the therapy.

**Procedures and treatment steps**
1. Clinical and radiological examination (panoramic image and CBCT)
2. Explanation of possible therapeutic approaches to the patient and the informed consent
3. Insertion of four mini-implants in the interforaminal region (open-flap surgery)
4. New panoramic image to control mini-implant positions
5. Sutures' removal
6. Control examination 7 days after sutures' removal; patient complaining of pain intensifying during tissue movements and chewing; movable mucosa of the cheek and lip on the right side with deep red inflammatory color; observation
7. Control examination after the next 7 days; the inflammation worsened, the mucous membrane was swollen, hard, indurated, and decubital injury was located opposing the MDI heads
8. The same day: a bulk of composite resin was placed around mini-implant heads and light-cured to prevent further injury
9. Next day: Clark's vestibuloplasty, suturing
10. Next day: a splint (in the form of occlusal rim) of high-viscosity silicone material made to cover MDIs, keep movable mucosa in place, and keep the medicament (Gengigel) for faster wound healing
11. 11 days later—custom impression (sutures in place)
12. 3 weeks after vestibuloplastic surgery, new dentures were finished and delivered; mini-implants were loaded through metal housings with “o” rings; movable mucosa was observed without any ulcerations and inflammation
13. Control examination and adjustments throughout 2 weeks
14. Control examination after 1 year; panoramic radiograph. - Healthy vestibular movable and attached mucosa—almost no peri-implant bone loss, increased patient satisfaction, and oral health-related quality of life

## Data Availability

All data in addition to those published are available from the authors on request.
